# The physician as person: The missing foundation in the CanMEDS roles

**DOI:** 10.36834/cmej.70280

**Published:** 2020-09-23

**Authors:** Lester Liao

**Affiliations:** 1 The Hospital for Sick Children, University of Toronto, Ontario, Canada

Physicians must take on many roles. The CanMEDS framework captures these nicely. But there is something that is more important than even the role of a medical expert, and that is the physician as person.^1^^,^^2^ This is easily forgotten in the muddle of clinical practice and training, but it is foundational. In the CanMEDS precursor form as the Educating Future Physicians of Ontario project, physician as person was one of eight key roles included for physicians.^3^ The fact that it was removed from updated CanMEDS roles suggests a shift in emphasis on what we believe physicianship to be at its core. Eerily, medicine has become depersonalized.

The physician has turned into a machine. From meeting endless patient demands in overcrowded ERs and swelling clinics to managing resources under the eye of the utilitarian, bureaucratic ethic, we have become cogs in a medical factory. We are unofficially reminded that our worth is in meeting health care system metrics. This has come at the cost of burnout and empathy decline.^4^^,^^5^ As humans, we crave significance and meaning in our work. When we are mechanized, we inevitably burn out. The soul atrophies and dies. This most certainly has not been intentional. Healthcare needs are pressing, but the unintended consequences of the relentless system on physicians has been heavy.

What we need is something that shifts our viewpoint at its very core. To do this well, we must consider the sociocultural influences that have brought us to our present predicament.

The educational system has unintentionally contributed to this process. Within Canada, the framework has evolved into Competency by Design for the Royal College, Triple C for the Family College, and competency based medical education across Undergraduate Medical Education and Continuing Professional Development. Evaluees are reminded that life is about check boxes. Even entrustable professional activities (EPAs) have morphed into a means to characterize residents as a whole, rendering their entire worth as a function of their work.^6^ The very proposal of CanMEDS roles was rooted in the language of outcomes.^7^ But patients need people, not CanMEDS machines.

There is a tendency in the medical world to be narrow in our focus. Many of us come from undergraduate backgrounds in the life sciences. We were cultured in a milieu that was generally indifferent toward the humanities. We then entered medical school and were inundated by information on the human body. Courses on the medical humanities or physicianship were ancillary, “soft,” and optional. These courses were completed to document progress, not humanity. This is not a surprise. There’s a lot of biomedical science to learn and clinical medicine to do. So, we focus ourselves on what we need to know to survive. But this has come at a cost. Our education has fostered a truncated vision of the world^8^^,^^9^ and of physicians.

This mindset has its roots in the development of instrumental rationalism. Over the past few centuries, science has become a means by which we manipulate the world for our benefit. Medicines alter bodily processes such that diseases are controlled and even cured. Gaining knowledge, then, has been about advancing this ability to control, about gaining power.^10^ This contrasts with the idea that knowledge helped people to order their lives wisely.^11^^,^^12^ While our new knowledge has been beneficial, the initial impulse of control has morphed into a life philosophy. This “instrumental reason” is mirrored in cost benefit analyses and maximizing output.^13^ Medicine is increasingly focused on technical means, and all problems, ranging from pain to existential suffering, are subjected to this apparatus. The physician is reduced to a catalyst in the production process.^14^ Combined with the idea that the human body is a machine, medicine has become a technical enterprise and physicians cold technicians.

But this philosophy was not without dissenters. The Romantic movement grew out of dissatisfaction with the sterility of the Enlightenment.^15^ Being human is more than manipulating machinery or applying logical proofs. Biochemical modulators and drug-induced happiness, as Huxley reminded us, does not solve the human predicament. Humans experience life more than they cogitate about it. Falling in love shapes us more than thinking about love ever could. We also see this in the way patients experience illness and encounter death. Physicians know this. We remember that medicine is a human enterprise. But especially under pressure, we are hopelessly amnestic creatures. If our humane reverie is not shattered by blinking phones or beeping pagers, the clinical deluge returns, and we slip back into robot mode, ploughing through the work until the day’s end. We become machines, forced into it by our unrelenting high- pressure work environments.

This trend has not gone by unnoticed. Physician wellness is a response to the fact that many physicians are burning out. Physicianship and professionalism courses remind us that what we do is far more than prescribe medicines and therapies. Narrative medicine taps into how stories make sense of our world.^16^ Palliative medicine and spirituality in medicine^17^ remind us that there is more to what we do than the technical science. We need to see what lies at the heart of these movements. It is that we are lost and need to re-ground ourselves in the idea that the doctor is first and foremost a person.

The physician as person must be highlighted, and we should acknowledge this formally in our CanMEDS roles, not as an additional role but as the very essence of a physician. This is not to ignore what we have gained and learned, nor is it to overlook societal or pragmatic concerns. It is to recognize both the finitude and vitality of the human condition. Ironically and unfortunately, we might then create techniques to increase outcome markers of humanity. But what we need is less to remind ourselves to *act* as people, to somehow take on the role of person, and instead we need an environment where we can *be nurtured as* people.^1^ We therefore need to reconsider how we conceive of medicine. The solution is not a minor alteration with another course or grand rounds tacked on. To drag it up from the world of technique and bureaucracy, we must think about resetting the foundation of medicine based on the physician as a person. Medicine is of people and for people, and we must reorder our profession around that.
